# SUMMER, a shiny utility for metabolomics and multiomics exploratory research

**DOI:** 10.1007/s11306-020-01750-7

**Published:** 2020-12-09

**Authors:** Ling Huang, Antonio Currais, Maxim N. Shokhirev

**Affiliations:** 1 Razavi Newman Integrative Genomics and Bioinformatics Core, The Salk Institute for Biological Studies, La Jolla, CA 92037 USA; 2 Cellular Neurobiology Laboratory, The Salk Institute for Biological Studies, La Jolla, CA 92037 USA

**Keywords:** Integrative analysis, Metabolomics, Multiomics, Bioinformatics software, Interactive user interface, Web server

## Abstract

**Introduction:**

Cellular metabolites are generated by a complex network of biochemical reactions. This makes interpreting changes in metabolites exceptionally challenging.

**Objectives:**

To develop a computational tool that integrates multiomics data at the level of reactions.

**Methods:**

Changes in metabolic reactions are modeled with input from transcriptomics/proteomics measurements of enzymes and metabolomic measurements of metabolites.

**Results:**

We developed SUMMER, which identified more relevant signals, key metabolic reactions, and relevant underlying biological pathways in a real-world case study.

**Conclusion:**

SUMMER performs integrative analysis for data interpretation and exploration. SUMMER is freely accessible at http://summer.salk.edu and the code is available at https://bitbucket.org/salkigc/summer.

**Electronic supplementary material:**

The online version of this article (10.1007/s11306-020-01750-7) contains supplementary material, which is available to authorized users.

## Introduction

With recent advances in untargeted metabolomics, thousands of steady-state metabolites can be profiled simultaneously in a standardized setting (Patti et al. 2012). After proper pre-processing and normalization, statistical analyses such as univariate/multivariate or regression analysis can identify significantly differentially expressed (DE) metabolites between a reference and a perturbed test condition (Cambiaghi et al. 2017). A wide range of tools have been developed to analyze the spectrum data, pre- and post-processing the data, and perform statistical analysis to find the DE metabolites (Forsberg et al. 2018; Spicer et al. 2017). However, functional interpretation of those DE metabolites within diverse biological contexts remains a significant challenge. Traditional pathway enrichment analysis assumes that most members of a given pathway are affected in response to a specific perturbation (Khatri et al. 2012). While this can work for well for global changes (Marco-Ramell et al. 2018), this assumption may not be useful for datasets with smaller changes or when only a part of the pathway is affected. In addition, traditional pathway enrichment analyses do not consider the interconnected nature of metabolites through metabolic reactions, thus they are not able to capture changes in a network occurring across multiple pathways or within a subnetwork of an annotated pathway. A reaction network approach is thus necessary to address these challenges.

Integration of metabolomics data with other types of omic high-throughput datasets, such as transcriptomics and proteomics, has been a popular topic in biological research. Several methods have been developed to integrate different types of omic data, such as overlap at the pathway level, correlation or partial correlation analysis, which identifies correlated changes across datasets, and integrated multivariate analysis, which looks for covariance associations between multiomics datasets (Cavill et al. 2016; Forsberg et al. 2018). However, these methods pose significant limitations. For example, they often consider transcripts and metabolites as independent observations and as such the correlated changes cannot be mechanistically interpreted. Other approaches try to integrate transcriptomics and proteomics into constraint-based models to predict flux distributions (Machado and Herrgard 2014). Only some of them have provided direct implementation, and often the implementation is in Matlab (Machado and Herrgard 2014), which is not freely available to users. Therefore, there is an urgent need for tools that are easily accessible and provide a functional readout of integrated multiomics datasets.

In this study, we have developed the Shiny Utility for Metabolomics and Multiomics Exploratory Research (SUMMER) to enable mechanistic interpretation of steady-state metabolomics data. SUMMER integrates metabolomics with enzyme activities estimated from transcriptomics or proteomics data by calculating changes in reaction rate potentials. The reaction rate potential is dependent on enzyme and substrate concentrations while inversely dependent on product concentrations. Thus, a reaction potential is designed to model the regulation of a reaction in a perturbed condition compared to a reference condition assuming that the ratio between substrates and products is balanced at the reference state. By looking for significant alterations in the ratio with correlated changes in enzyme activity, SUMMER is able to identify the hotspot reactions of a perturbed system. After the change in rate potentials is estimated, SUMMER visualizes the network graph of all significant metabolic changes and runs pathway enrichment analysis for data exploration and functional interpretation. Our simulation study shows that the integration of the enzyme activities significantly improved the accuracy of estimating the change in reaction rate potentials compared to a non-integrated model. To demonstrate its utility, particularly for datasets with weak metabolic changes, we apply SUMMER to a case study to reveal underlying biological signals. SUMMER has a user-friendly web interface for researchers to upload their data, perform quality checks and quantitative analyses, and interactively explore outputs. SUMMER was developed in the R shiny framework (Chang et al. 2018) and it is freely accessible at http://summer.salk.edu and https://bitbucket.org/salkigc/summer/.

## Materials and methods

### Data preprocessing

Total metabolic reactions and their related compounds, enzymes, and pathways information were downloaded from the Kyoto Encyclopedia of Genes and Genomes (KEGG) (Kanehisa et al. 2019) REACTION database through the KEGG REST API.

For each new job query, data is first mapped to KEGG identifiers. Metabolites or proteins with more than half of the values missing are removed from downstream analyses. We further assume that any remaining missing values represent concentrations that were too low to be detected, and impute them based on the minimal value across samples. After that, quantile normalization is applied for spectrum data and microarray fluorescent data to remove systemic technical bias. Next, transcripts or protein abundances are aggregated to calculate the catalytic enzyme activity by incorporating the information about isoenzyme and enzyme subunits available from the KEGG database. The enzyme activity is set to the minimal abundance among measured subunits and the sum of all isoenzyme abundances. All data is then log2-transformed after adding a pseudo count of 1. The top 10% most variable transcripts/proteins and total metabolites are used for principle component analysis (PCA), which provides a global summary of the data.

### Quantitative analysis of metabolomics data

To determine fold changes in metabolites, a univariate test is performed for metabolites by limma (Ritchie et al. 2015) with default settings. A robust regression model is used for datasets with more than 5 biological replicates. Significant changes are defined to have a multiple testing adjusted p-value < 0.05 and absolute log_2_ fold-change (log2FC) > 0.5.

### Integration of metabolomics data with transcriptomics or proteomics data through metabolic reaction rate potentials estimation

Reaction rate potentials at steady-state were formulated to be positively correlated with enzyme and substrate concentrations and negatively correlated with product concentrations to model feedback regulatory effects (Vital-Lopez et al. 2013):

$$r=k[p]\frac{{\prod }_{i}{[{A}_{i}]}^{{m}_{i}}}{{\prod }_{j}{[{B}_{j}]}^{{m}_{j}}}$$

In the equation, r represents the reaction rate; k is reaction-specific rate coefficient inherent to enzyme p for reaction r; A denotes substrates, B denotes products, and m is an approximate stoichiometric coefficient for the reaction (m = 1 for stoichiometric coefficient of 1 and m = 2 otherwise); i and j refer to individual components of the metabolic reaction.

Assuming unchanged rate coefficient k (enzyme catalytic nature unchanged between states), fold-change of reaction rate potentials between a reference state and a test state can be estimated by combining changes in substrates, products, and enzyme between test and reference states:

$${r}^{test}/{r}^{ref}= {([p}^{test}]{\prod }_{i}{{{[A}_{i}}^{test}/{{A}_{i}}^{ref}]}^{{m}_{i}})/{([p}^{ref}]{\prod }_{j}{{{[B}_{j}}^{test}/{{B}_{j}}^{ref}]}^{{m}_{j}})$$

The log_2_ fold-change of reaction rate potential between a test state and a reference state can be shown as:

$${\text{log}}_{2}\text{FC}\left(r\right)= {\text{log}}_{2}({r}^{test}/{r}^{ref})$$

Then the equation can be expressed in terms of sums as:

$${\text{log}}_{2}\text{FC}\left(r\right)={\text{log}}_{2}\text{FC}\left(p\right)+{\sum }_{i}{{\text{log}}_{2}\text{FC}\left({A}_{i}\right)*m}_{i}- {\sum }_{j}{{\text{log}}_{2}\text{FC}\left({B}_{j}\right)*m}_{j}$$

The change in reaction rate potentials indicates deviation of the reactions compared to a reference state and represents the potential of the reaction to return back to the reference state. A positive change of rate potential suggests that the biochemical reaction will consume more substrates and produce more products to keep the balance between products and substrates. In other words, low product abundance and high substrate abundance will have a high reaction rate potential. Only expressed reactions with at least one measured substrate and one measured product are tested. A bootstrap approach involving 500 random samplings of metabolites and enzyme fold-changes is applied to infer the range of possible fold-changes in a particular reaction rate potential, which is a similar approach to the gene bootstrapping option provided by GSEA (Subramanian et al. 2005). Briefly, metabolites and enzymes concentration changes are sampled randomly to obtain a range of possible fold-changes in reaction rate potentials that is then used to calculate the ranking score (ranging from 0 to 1) for each reaction. A smaller ranking score indicates a higher ranking of absolute fold-changes of reaction rate potentials compared to possible changes estimated from random background of the input dataset. Reactions with a ranking score < 0.05 and absolute log2FC > 0.5 are identified as significant.

### Pathway analysis and network graph

In addition to a network-based analysis, SUMMER also performs a standard over-representation analysis on the DE metabolites and DE reactions using WebGestaltR (Wang et al. 2017) to identify significantly altered KEGG pathways. In the pathway view of the network graph, all measured metabolites will be shown regardless of their statistical significance. In the global view of the network graph, only DE metabolites and DE reactions plus their associated reactions and measured metabolites will be shown.

### Simulation

The mean of biological replicates was simulated using gamma distribution with parameters estimated from the metabolomics and transcriptomics datasets previously generated from brain samples of SAMP8 mice (Currais et al. 2019). The standard deviation of biological replicates was simulated using a decay distribution relative to the mean to reflect the fact that sample variation decreases with mean as what was observed from the SAMP8 datasets. Distribution parameters were manually adjusted based on observations. Six biological replicates were then generated using a normal distribution for the log-transformed data. 10% of the reactions (5% up and 5% down) were randomly selected from total reactions to represent truly changed reactions. Their associated enzymes and metabolites were set as truly changed enzymes and metabolites accordingly. When there was a conflict in the direction of change for a metabolite, which can happen if a metabolite participates in multiple reactions, it was set as unchanged. A log2FC of 0.01, 0.1, 0.2, 0.3 was added to the mean when simulating the truly changed data. Receiver Operating Characteristic (ROC) curves were used to evaluate the performance of simulated data using package “pROC” (Robin et al. 2011).

## Results and discussion

### Workflow

**Fig. 1:**
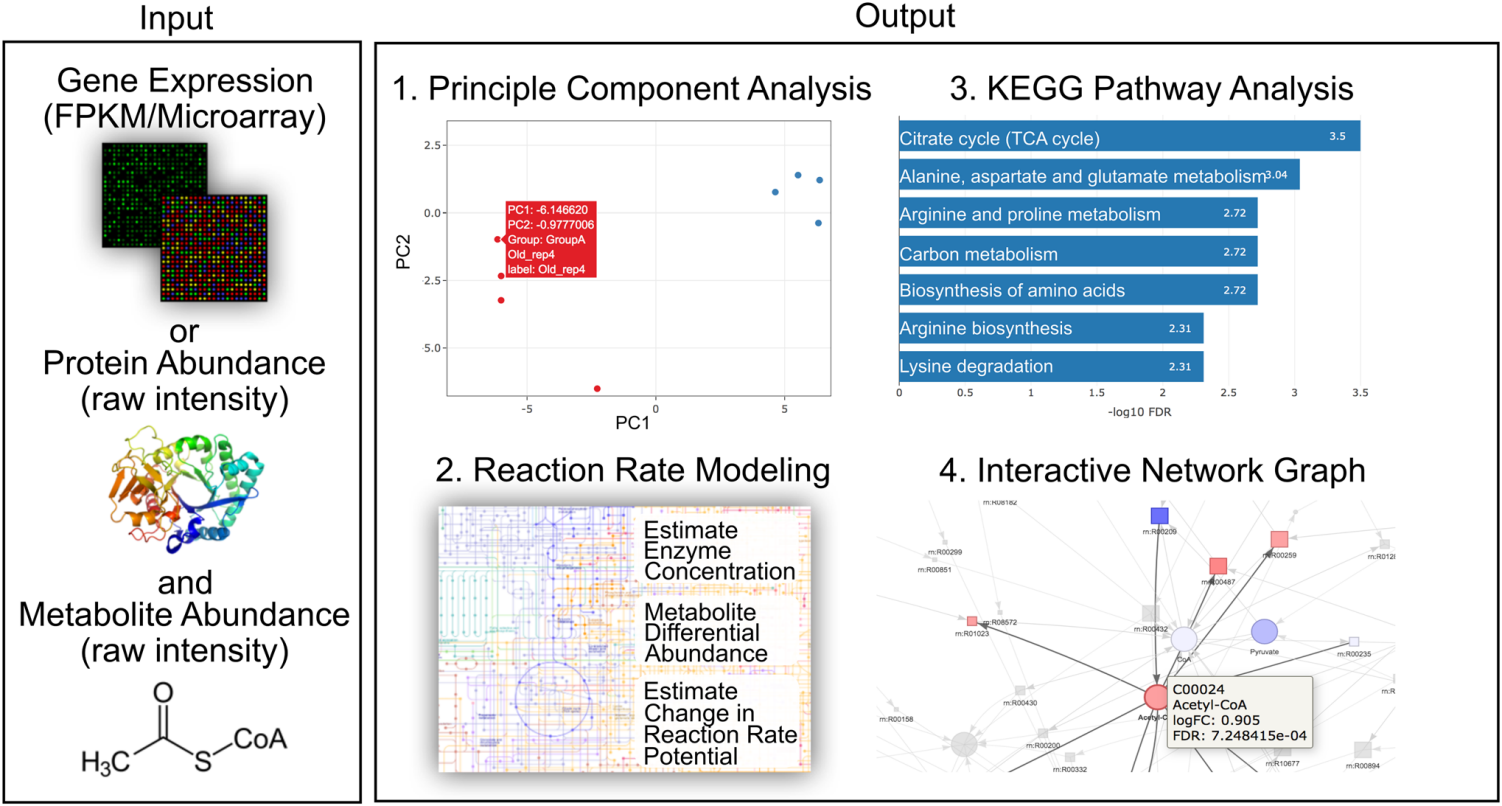
SUMMER overview. Gene expression or protein abundance, and metabolite abundance measurements across two conditions are used to check for overall signal separation with principal component analysis and then integrated using KEGG reactions. SUMMER estimates enzyme concentration from protein or gene expression data, identifies changes in metabolite levels, and estimates changes in reaction rate potentials. Results can then be interpreted using interactive network graphs with colors indicating changes, and KEGG enrichment analysis to identify changes in pathways

Figure 1 illustrates a typical SUMMER workflow. SUMMER requires metabolite raw intensity and either gene or protein abundances as input for enzyme activity estimation. After mapping the input entries to the Kyoto Encyclopedia of Genes and Genomes (KEGG) (Kanehisa et al. 2019) identifiers, SUMMER performs a principle component analysis on all input datasets for the users to visualize signal separation. Differential expression (DE) analysis on the metabolites is carried out by “limma” (Ritchie et al. 2015) using linear modeling and empirical Bayes posterior variance estimators. DE analysis on the reactions is estimated by bootstrap strategy. An over-representation analysis to identify significantly enriched KEGG pathways terms is then carried out on DE metabolites and DE reactions by “WebGestaltR” (Wang et al. 2017). A network of metabolites and reactions annotated by their statistical significance for each specific KEGG pathway can be generated based on the user’s choice. Finally, a global network graph connected by DE metabolites and reactions is constructed for users to visualize the overall changes. All graphs and visualizations are shown in an interactive way that allows users to better explore the data. The results can be downloaded and the network can be visualized in Cytoscape (Shannon et al. 2003). The current implementation of SUMMER utilizes paralleled computing to efficiently perform bootstrap testing in a few minutes but limiting the use of the tool to one user at a time.

### Simulation

**Fig. 2:**
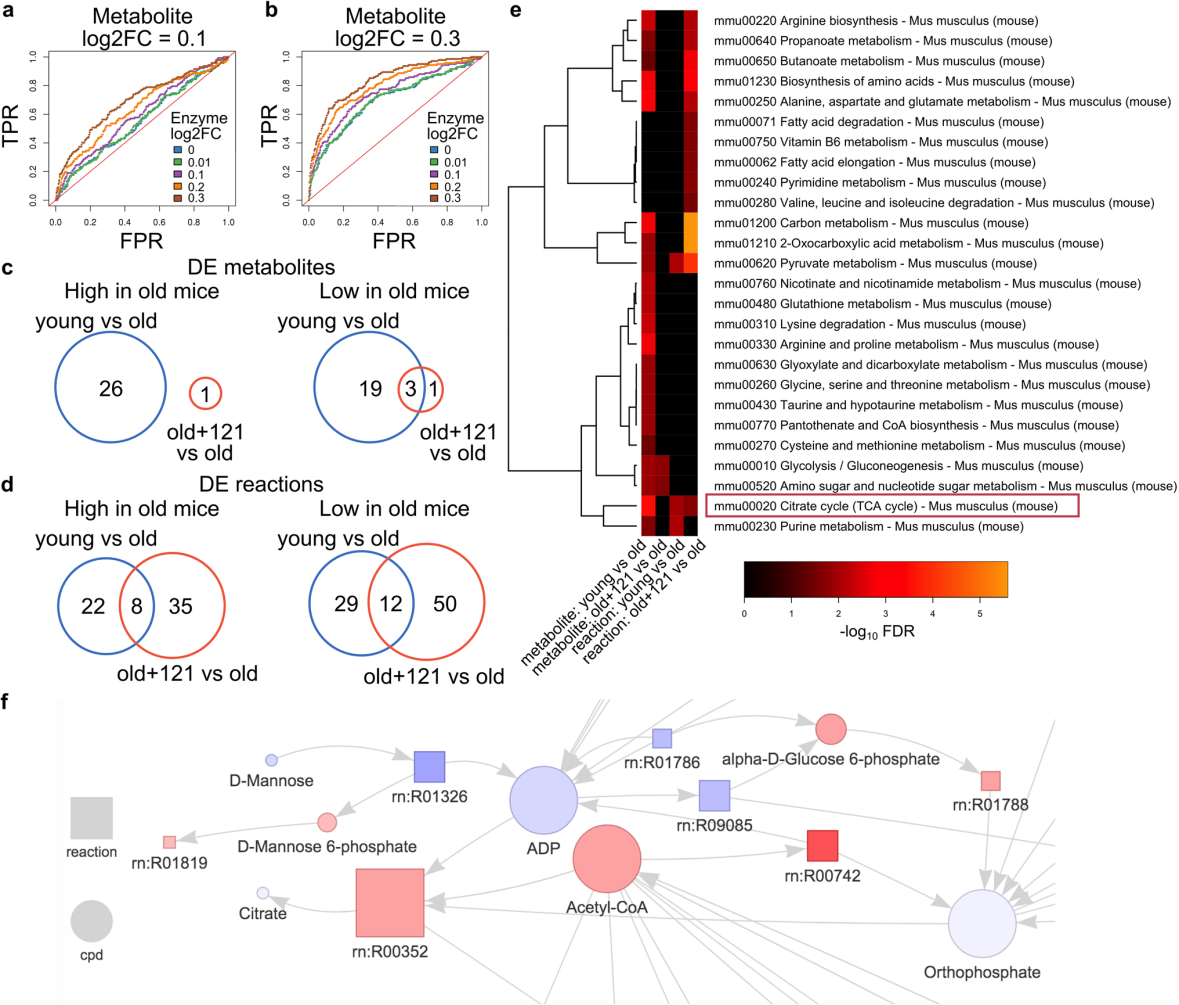
SUMMER helps to reveal biological signals. **a**, **b** Receiver Operating Characteristic (ROC) curve of simulated data with log_2_ fold-change (log2FC) = 0.1 (**a**) and 0.3 (**b**). *FPR* false positive rate, *TPR* true positive rate. **c** Venn diagram of overlapping Differentially Expressed (DE) metabolites between two tests: young vs old and old + 121 versus old. **d** Venn diagram of overlapping DE reactions between two tests: young vs old and old + 121 versus old. **e** Heatmap of significantly enriched KEGG pathways terms identified using DE metabolites/reactions of the two tests. *FDR* false discovery rate. **f** Network connected by the three common DE metabolites identified by two tests. Data presented using old + 121 versus old dataset. The interactive form of the whole network is included in Supplemental File 1. Color intensity indicates log2FC, *red* up-regulation, *blue* down-regulation, node size represents node connectivity, *cpd* compound (metabolite)

In order to assess how the integration of enzyme data affects the estimate of reaction rate potentials, 10% of total reactions associated metabolites and enzymes were simulated to have a log_2_ fold-change (log2FC) of 0.01, 0.1, 0.2, and 0.3. A Receiver Operating Characteristic (ROC) curve was used to compare the Area Under the Curve (AUC). As expected, metabolites with greater log2FC led to higher AUC of rate potential estimates when the enzyme data was not available (compare Fig. 2a, b, blue lines). However, when integrated with enzyme data, the AUC was improved with the increase of enzyme log2FC (Fig. 2a, b, colored lines, see also Supplemental Dataset1). When metabolite log2FC was set at 0.3 (Fig. 2b), the integration of enzyme data at log2FC = 0.1 and log2FC = 0.3 improved the AUC from 0.699 to 0.749 (7%) and 0.836 (20%), respectively (Supplemental Dataset 1).

### Case study

In this case study, we re-analyzed the effects of a neuroprotective compound in the SAMP8 mouse model of accelerated aging and dementia using metabolomics and transcriptomics datasets prepared from brain tissue of these animals (Currais et al. 2019). It was previously shown that SAMP8 mice developed significant deficits in brain function and physiology with age that were prevented by two neuroprotective compounds, in particular CMS121. The old SAMP8 mice treated with CMS121 (old + 121) were found to have a younger phenotype compared to old untreated control mice in terms of cognitive function and transcriptional signatures (Currais et al. 2019). It was questioned whether drug-treated mice would have metabolic commonalities with the young mice. To address this, we compared the DE metabolites altered between old + 121 and old mice with those altered between young and old mice. However, a standard differential metabolite analysis did not reveal many similarities between the two comparisons (Fig. 2c, Supplemental Dataset 2). When the data was analyzed by SUMMER using the integrated model that considers both metabolomics and transcriptomics data, we found 20 correlated DE reactions (8 up- and 12 down-regulated in old untreated mice) that showed the same direction of change shared by the two comparisons (Fig. 2d, Supplemental Dataset2). This overlap in DE reactions demonstrates the values of an integrative network-based approach. Importantly, a critical reaction rn:R00742 (ATP + Acetyl-CoA + HCO3- <  =  > ADP + Orthophosphate + Malonyl-CoA) was among the 20 correlated DE reactions identified by both old + 121 versus old and young vs old comparisons (Supplemental Dataset 2, See also Fig. 2f). This reaction is catalyzed by the enzyme acetyl-CoA carboxylase (ACC1), which we previously found to be a molecular target of CMS121 critical to maintaining a younger metabolic phenotype (Currais et al. 2019). For this particular reaction, SUMMER identified it as being up-regulated in young and drug-treated mice compared to the reference state in old mice because the concentrations of both the enzyme activity and substrate acetyl-CoA were up-regulated, which suggested that a higher reaction rate is needed in young and drug-treated mice for the substrates and products to “return back” to the old mice state. Indeed, if the drug is removed from the system and the enzyme function is not preturbed, we would expect to see a rapid increase of the reaction rate in the drug-treated mice. SUMMER identified an important link in acetyl-CoA metabolism that was affected in old mice and reverted by the drug treatment.

We also found before that the tricarboxylic acid (TCA) cycle was profoundly altered with aging in SAMP8 mice, which was partially prevented by the treatment with CMS121 (Currais et al. 2019). Pathway enrichment analysis based on DE metabolites and DE reactions of young vs old mice confirmed the TCA cycle to be one of the significantly enriched KEGG terms (Fig. 2e, column 1 and column 3). On the other hand, pathway results based on just the DE metabolites of old + 121 versus old dataset did not show these signals (Fig. 2e, column 2) whereas pathway result based on DE reactions were able to capture the TCA cycle as a significant biological signal for the drug treatment (Fig. 2e, column 4). This demonstrates that the reaction potential changes estimated by SUMMER were associated with real underlying metabolic changes.

Finally, SUMMER provides an interactive network graph that allows data exploration and functional interpretation. The three common DE metabolites shared between young vs old and old + 121 versus old were alpha-D-Glucose 6-phosphate, acetyl-CoA, and D-Mannose 6-phosphate. However, the three metabolites do not overlap at the pathway level except for the two very general pathways: Metabolic Pathways, and Biosynthesis of Secondary Metabolites. The interactive network graph constructed by SUMMER helped to reveal the relationship between the three metabolites because they were all connected through ADP and orthophosphate (Fig. 2f, Supplemental File 1). This suggests the potential role of ADP and orthophosphate in the metabolic network of drug-treated samples even if they were not themselves significantly changed. Since we used steady-state levels of metabolites, we don’t have information on their turnover rates. It is possible that the turnover rates of ADP and orthophosphate were significantly altered in young and drug-treated mice compared to the old mice, which would suggest a rewire of the metabolic flow as a consequence of an altered TCA cycle. Interestingly, the key reaction rn:R00742 was identified as one of the few reactions connecting the three common DE metabolites through ADP and orthophosphate. The inhibition of ACC1 led to a build-up in acetyl-CoA levels and a decrease in fatty acid biosynthesis (Currais et al. 2019), representing a key step in the rewiring of the metabolic flow. Combined together, the network graph connects the key metabolites and reactions critical to the perturbed metabolic network compared to a reference state, providing novel insights for observed data and allowing for the generation of new hypothesis based on the backbone of metabolic network models and mathematical modeling.

SUMMER assumes that the input intensity values reflect the actual analyte concentration in the sample and uses them to calculate the fold-change and related change in reaction rate potentials. However, the resulted fold-change might be underestimated or overestimated (“fold-change compression”) due to the nonlinear signal response of electrospray ionization (Yu et al. 2020). Therefore, we suggest using a quality control sample-based signal calibration workflow to calibrate the intensity values prior to the SUMMER workflow.

SUMMER was designed to detect changes in reaction rate potentials comparing a perturbed condition to a reference condition, assuming the reaction has a balanced ratio of substrates to products at a reference state. It is dependent on the changes in enzyme abundances and metabolites abundances. Therefore, it is not able to detect changes in cases with increased flux but no deviation of the ratio or when the entire pathway is changing in the same direction. Metabolic flux analysis, such as that provided by isotope tracing, would be required for further clarification. SUMMER focuses on the reactions that are significantly altered in the ratio of substrates and products, which might serve as hotspots of a given perturbed system. Our case study based on the preventive effects of CMS121 on aging mice illustrates the power of using SUMMER to identifying the underlying key events.

## Concluding remarks

SUMMER is a free, complete multiomics analysis platform that incorporates a variety of modules including data cleaning and filtering, multivariate analysis, DE analysis, pathway analysis, and network analysis to facilitate the biological interpretation of high-throughput multiomics datasets. It provides a graphic interface that allows broad use by the scientific community including a maintained online server and open sourced code. SUMMER is designed to help researchers to integrate multiomics datasets, visualize and explore the data for results interpretation and hypothesis generation.

## Electronic supplementary material

Below is the link to the electronic supplementary material.

Electronic supplementary material 1 (XLSX 53 kb). Supplemental Dataset 1. Area Under the Curve for simulated metabolites and enzymes at different log2 Fold-Change

Electronic supplementary material 2 (XLSX 392 kb). Supplemental Dataset 2. Differential Expression analysis results and pathway enrichment results computed by SUMMER for metabolites and reactions of young vs old and old+121 vs old comparisons. Only metabolites mapped to KEGG identifiers were included in the results

Electronic supplementary material 3 (HTML 1311 kb). Supplemental File 1. Interactive network graph constructed for old+121 vs old comparison by SUMMER using default cutoff log2FC > 0.5, FDR < 0.05, and ranking score < 0.05

## Data Availability

The source code can be obtained at https://bitbucket.org/salkigc/summer/ under the MIT license. The online server is freely accessible at http://summer.salk.edu. The data re-analyzed by this study was from (Currais et al. 2019) with GEO accession number GSE101112 and also available at SUMMER web server as sample datasets.
